# *Lysobacter gummosus* 10.1.1, a Producer of Antimicrobial Agents

**DOI:** 10.3390/microorganisms11122853

**Published:** 2023-11-24

**Authors:** Irina Kudryakova, Alexey Afoshin, Sergey Tarlachkov, Elena Leontyevskaya, Natalia Suzina, Natalia Leontyevskaya (Vasilyeva)

**Affiliations:** Laboratory of Microbial Cell Surface Biochemistry, G.K. Skryabin Institute of Biochemistry and Physiology of Microorganisms, FRC PSCBR, Russian Academy of Sciences, 5 Prosp. Nauki, Pushchino 142290, Russia; kudryakovairina@yandex.ru (I.K.); alex080686@mail.ru (A.A.); sergey@tarlachkov.ru (S.T.); ealeont@gmail.com (E.L.); suzina_nataliya@rambler.ru (N.S.)

**Keywords:** *Lysobacter*, antimicrobial agents, bacteriolytic enzymes, RNA-seq, secondary metabolites

## Abstract

This work investigated the antimicrobial potential of *Lysobacter gummosus* 10.1.1. The culture fluid of the strain was found to contain antimicrobial agents active against *Staphylococcus aureus*, *Micrococcus luteus*, and *Bacillus cereus*. *L. gummosus* was first shown to be capable of forming outer membrane vesicles, which have a bacteriolytic effect against not only Gram-positive bacteria but also against the Gram-negative pathogen *Pseudomonas aeruginosa*. Transcriptomic analysis revealed the genes of almost all known bacteriolytic enzymes of *Lysobacter*, as well as the genes of enzymes with putative bacteriolytic activity. Also identified were genes involved in the biosynthesis of a number of secondary metabolites for which antimicrobial activities are known. This research is indicative of the relevance of isolating and studying *L. gummosus* antimicrobial agents.

## 1. Introduction

When Christensen and Cook formed the genus *Lysobacter* in 1978, it included four species—*L. gummosus*, *L. brunescens*, *L. enzymogenes*, and *L. antibioticus*—with antagonistic activities against various, including pathogenic, bacteria and fungi [1]. Over 50 species of *Lysobacter* are known to date. Lytic activities, however, have been shown for only five species of the genus: the species *L. capsici* has been added to the first four. These lytically active *Lysobacter* species have been shown to be capable of producing various antimicrobial compounds: antibiotics, short peptides, and bacteriolytic enzymes [2,3,4,5,6,7]. The most investigated are *L. capsici* and *L. enzymogenes*. The profiles of antibiotic compounds have been studied for both species, especially for *L. enzymogenes* [2,3,5,8,9,10,11]. Cells of *L. capsici* and *L. enzymogenes* are capable of forming outer membrane vesicles (OMVs) that contain antibiotics and/or bacteriolytic enzymes. This significantly expands the antimicrobial potential of these species [4,12,13].

Our scientific interest is in the search, isolation, and characterization of secreted bacteriolytic enzymes of *Lysobacter* bacteria. The substrate for these enzymes is the peptidoglycan of competitive bacteria. By specificity of their action against peptidoglycan, they are bacteriolytic proteases, amydases, and glycosidases (muramidases and N-acetylglucosaminidases). Such a wide spectrum of action against peptidoglycan determines the relevance of the search for and study of these enzymes in order to create, based on them, antimicrobial drugs for the treatment of infections caused by antibiotic-resistant pathogenic strains.

The known bacteriolytic enzymes of *L. enzymogenes* are α- and β-lytic proteases and amidase CwhA [14,15]. Bacteriolytic enzymes of *L. capsici* species of strains XL1 and VKM B-2533^T^ are the object of our research. Isolated and characterized to varying degrees to date are bacteriolytic proteases Blp and L1 homologous to α- and β-lytic proteases of *L. enzymogenes*; proteases L4, L5, Serp, Serp3, Serp6, and Serp7; amidase L2; and N-acetylglucosaminidase [6,7,16,17]. Our transcriptomic study of *L. capsici* VKM B-2533^T^ has revealed the significant antimicrobial potential of this species and has allowed us to find and isolate new bacteriolytic enzymes [7]. This approach has met our expectations in the search for the genes of antimicrobial agents. In the present paper, we set ourselves the task of studying the antimicrobial potential of *L. gummosus*, one of the first four species included in the genus *Lysobacter*.

To date, *L. gummosus* has been established to possess chitinase, protease, and glucanase activities, as well as a lytic activity against *Arthrobacter* sp., *Bacillus subtilis*, *Actinomycete* UASM 4432, *Actinomycete* UASM 4441, *Xanthomonas campestris* pv. *campestris*, *Pectobacterium atrosepticu* bacteria, biofilms of *Staphylococcus epidermidis*, and a number of fungi and algae [1,18,19]. MALDI-TOF has identified several proteins in the culture fluid of *L. gummosus*-type strain DSMZ 6980: α- and β-lytic proteases, two lysine-specific proteases, hemagglutinin/proteinase, OmpA-like protein 1, and OmpA-like protein 2 [18]. Not a single lytic agent has been isolated, however. Strain *L. gummosus* 10.1.1 has been isolated from suppressive soils in the Netherlands [20]. The strain has been shown to possess antibacterial and antifungal activities [19]. We have recently sequenced the DNA of *L. gummosus* 10.1.1 and have assembled its genome in the form of a ring chromosome [21]. The present work continues the characterization of this strain, a potential producer of valuable antimicrobial agents.

## 2. Materials and Methods

### 2.1. Cultivation Conditions

Media of the following composition (g/lL) were used for cultivation of strain *L. gummosus* 10.1.1: RM medium: glucose (PanReac AppliChem, Barcelona, Spain), 5.0; peptone (Diaem, Moscow, Russia), 2.0; yeast extract (Helicon, Moscow, Russia), 2.0; Na_2_HPO_4_·12H_2_O (PanReac AppliChem, Barcelona, Spain), 4.2; KH_2_PO_4_ (Helicon, Moscow, Russia), 1.0; KCl (PanReac AppliChem, Barcelona, Spain), 0.6; MgSO_4_·7H_2_O (PanReac AppliChem, Barcelona, Spain), 5.0; pH 7.0 [22]; 5/5 medium developed at the IBPM RAS: yeast extract (Helicon, Moscow, Russia), 1.0; soybean extract (Helicon, Moscow, Russia), 30.0; tryptone (Helicon, Moscow, Russia), 5.0; amino peptide (Samson-Med, St. Petersburg, Russia), 60.0, pH 7.2. Strain *L. capsici* VKM B-2533^T^ was cultivated on RM medium. Cells of both strains were cultivated for 19 h at 29 °C with stirring (205 rpm). Bacterial test objects *Staphylococcus aureus* 209P, *Micrococcus luteus* Ac-2230^T^, *Kocuria rosea* Ac-2200^T^, *Bacillus cereus* 217, *Proteus vulgaris* H-19, and *Pseudomonas aeruginosa* were cultivated for 18 h on 5/5 medium with the addition of 1.5% agar. Fungal test objects *Fusarium solani* and *Sclerotinia sclerotiorum* were cultivated for 48 h on wort medium with the addition of 1.5% agar at 29 °C. Cell biomass of the test objects was obtained by flushing from the cultivation medium with a buffer of 10 mM Tris-HCl, pH 8.0.

### 2.2. *Turbidimetric* Determination of Bacteriolytic Activity

Cells of the test objects were washed twice in 10 mM Tris-HCl, pH 8.0, via centrifugation at 7000× *g* for 10 min on a D2012 plus centrifuge (DLAB Scientific, Beijing, China). The same buffer was added to the washed cells; absorption of the cell suspension was adjusted to OD_600_ = 0.5. The reaction mixture contained 950 μL of the cell suspension and 50 μL of the preparation *of L. gummosus* 10.1.1 or *L. capsici* VKM B-2533^T^ culture fluid. The mixture was incubated at 37 °C for 5–10 min. The reaction was arrested by placing test tubes in ice. The bacteriolytic activity (LU/mL) was calculated by the following formula:

[0.5 (initial OD_600_ of the suspension) − final OD_600_] × 1000 μL (total reaction volume)/[min (time of reaction) × μL (volume of sample) × 0.01 (correction coefficient for the OD reduction per min)]

The measurements were carried out in two biochemical repeats for each of the three biological experiments.

### 2.3. Determination of Lytic Activity via Spot Test

*S. aureus* 209P, *M. luteus* Ac-2230^T^, *K. rosea* Ac-2200^T^, *B. cereus* 217, *P. vulgaris* H-19, and *P. aeruginosa* bacterial cells with OD_600_ of 3.4, 6.8, 3.9, 3.6, 2.2, and 1.6, respectively, and *F. solani* and *S. sclerotiorum* fungal cells with OD_600_ of 0.8 each were inoculated into Petri dishes with agarized medium 5/5 and wort, respectively. The dishes were incubated for 1 h at 29 °C. To assay their bacteriolytic activities, preparations of culture fluid and vesicles were applied in amounts of 10 and 30 μL, respectively, on the lawn of target cells. To determine the antifungal activities, wells were made in agar, into which 25 μL of preparations each was added. The dishes were incubated at 29 °C for 24–36 h. The emergence of a lysis zone at the site of application testified to the presence of antimicrobial agents. A transparent lysis zone was indicative of a strong lytic effect of the preparation (++). The turbid lysis zone meant a weak lytic effect (+). A confluent growth of test culture cells at the site of application indicated the absence of lytic action (–). To determine the lytic activities of vesicles, we used preparations from two independent biological experiments.

### 2.4. Isolation of Outer Membrane Vesicles

Cells of *L. gummosus* 10.1.1 and *L. capsici* VKM B-2533^T^ were cultivated for 19 h at 29 °C in 700 mL shaken flasks containing 150 mL of liquid RM medium. Then, the culture fluid was freed of cells via centrifugation at 7000× *g* for 20 min on an Avanti J-26XP centrifuge (Beckman Coulter, Brea, CA, USA). Vesicles were precipitated from 180 mL of culture fluid by centrifugation at 113,000× *g* for 2 h on an L7-55 ultracentrifuge (Beckman Coulter, Brea, CA, USA). The precipitate was washed twice in 50 mM Tris-HCl, pH 8.0, by centrifugation at the same speed. At the last stage, 200 µL of 50 mM Tris-HCl, pH 8.0, was added to the vesicle precipitate, the contents were mixed, and the resulting preparations were used for analysis.

### 2.5. Protein Concentration Assay

Protein concentration was determined in preparations of *L. gummosus* 10.1.1 and *L. capsici* VKM B-2533^T^ culture fluid using a BCA kit (FineTest, Wuhan, China). Samples for analysis were prepared as follows: 5 mL of the strains’ culture fluid was treated with TCA to a concentration of 10% in solution, and the mixture was incubated at 4 °C for 3 h. Then, the mixture was centrifuged at 25,000× *g* for 10 min on an Avanti J-26XP centrifuge (Beckman Coulter, Brea, CA, USA) to precipitate proteins. The resulting protein precipitates were washed twice with cold concentrated acetone by centrifugation at the same speed. Then, the precipitates were dried, and 130 µL of 50 mM Tris-HCl, pH 8.0, containing 5% SDS, was added. The further procedures were according to the BCA kit manufacturer’s instructions. The concentration of protein in the preparations was determined by a calibration curve plotted for BSA dissolved in 50 mM Tris-HCl, pH 8.0, with 5% SDS, within the concentration range from 0.25 to 2.00 mg/mL.

### 2.6. Transmission Electron Microscopy

Vesicle samples of *L. gummosus* 10.1.1 and *L. capsici* VKM B-2533^T^ were placed on top of a formvar-coated copper grid. The applied sample was allowed to adsorb for 2 min, and sample excess was then removed using filter paper. After air drying, the samples were stained with a 0.3% aqueous solution of uranyl acetate (pH 4.0), placed on the grids, and immediately removed using filter paper. Negatively stained preparations were examined with a JEM-1400 transmission electron microscope (JEOL, Tokyo, Japan) at an accelerating voltage of 80 kV, and random images of representative fields of observation were captured with a MORADA G2 11-megapixel TEM camera (EMSIS GmbH, Münster, Germany).

### 2.7. SDS-PAGE under Denaturing Conditions

The electrophoresis was performed in 12.5% PAG in the presence of sodium dodecyl sulfate according to Laemmli [23]. The protein profiles were compared by taking 12 µL of the culture fluid of *L. gummosus* 10.1.1 and *L. capsici* VKM B-2533^T^, which corresponded to the sample’s protein content of 0.25 µg and 0.5 µg, respectively. The samples were heated in a sample buffer (0.025 M Tris-HCl, 2% SDS, 10% glycerol, 0.7 M mercaptoethanol, bromophenol blue, pH 6.8) at 99 °C for 10 min. As markers, a mixture of protein standards (Thermo Fisher Scientific, Waltham, MA, USA) was used: β-galactosidase, 116.0 kDa; BSA, 66.2 kDa; ovalbumin, 45.0 kDa; lactate dehydrogenase, 35.0 kDa; REase Bsp981, 25.0 kDa; β-lactoglobulin, 18.4 kDa; lysozyme, 14.4 kDa. The electrophoresis in the concentrating gel was performed at 90 V; in the separating gel, at 180 V. Protein bands in the gel were revealed by staining with imidazole and ZnCl_2_ solutions [24].

### 2.8. Isolation of RNA

Cells of *L. gummosus* 10.1.1 were cultivated in RM and 5/5 liquid media at 29 °C for 19 h in three biological repeats. Then, 500 µL of the culture was taken from each flask and centrifuged at 7000× *g* for 10 min on a D2012 plus centrifuge (DLAB Scientific, Beijing, China). Next, the biomass was broken down using ice-cold zirconia beads in a 0.5 mL screw cap tube, and the RNA was isolated using a RiboPure RNA Purification Kit (Thermo Scientific, Waltham, MA, USA) in accordance with the manufacturer’s recommendation. The concentration of the RNA in the preparations obtained was measured on a NanoDrop OneC device (Thermo Fisher Scientific, Waltham, MA, USA). The quality of the RNA preparations was assessed electrophoretically in 4% PAG with 8 M urea as well as by capillary electrophoresis using a Bioanalyzer 2100 (Agilent, Santa Clara, CA, USA). Ribosomal RNA was removed using a Ribo-Zero Plus rRNA Depletion Kit (Illumina, San Diego, CA, USA). cDNA synthesis with the subsequent preparation of libraries was carried out using a NEBNext Ultra II Directional RNA Library Prep Kit for Illumina (New England Biolabs, Ipswich, MA, USA). The library was sequenced on the Illumina HiSeq 4000 system (Illumina, San Diego, CA, USA) to obtain 151 bp reads.

### 2.9. RNA-Seq Data Analysis

The quality of the reads was controlled using FastQC v0.12.1 [25]. Adapter sequences and low-quality regions in raw reads were removed using Trimomatic v0.39 [26]. Clean reads were mapped on the *L. gummosus* 10.1.1 genome (GenBank access No. CP093547.1) using the Bowtie2 program v2.5.1 [27]; the mapped reads were counted using the featureCounts v2.0.4 [28]. The DESeq2 v1.34.0 package was used to assess differential gene expression [29]. Medium 5/5 was used as a control. A gene was assumed to change expression level if adjusted *p*-value (*p*_adj_) < 0.05. The phylogenomic tree was inferred using JolyTree v2.1.211019ac [30]. NRPS/PKS clusters in *Lysobacter* genome sequences were identified using the antiSMASH 7.0.0 database [31].

### 2.10. Statistical Analysis

Statistical analysis was performed using GraphPad Prism version 8.0.1 (GraphPad Software, San Diego, CA, USA). All experiments were conducted with at least three repeats. The data are presented as means ± standard deviations, as well as in the form of boxplots (medians ± interquartile spans). The data were considered to be significant at *p* < 0.05. The normal distribution of the data was verified using the Shapiro–Wilk test. To determine the equality of the variances of two independent groups, the *F*-test was used. For the normally distributed data of two groups, the two-sided unpaired Student’s *t*-test was used; for other data types, the two-sided Mann–Whitney *U*-test was applied.

## 3. Results

### 3.1. Characterization of Strain L. gummosus 10.1.1

The strain *L. gummosus* 10.1.1 was a kind gift from Dr. Joeke Postma (Wageningen University and Research Centre, The Netherlands).

**Figure 1:**
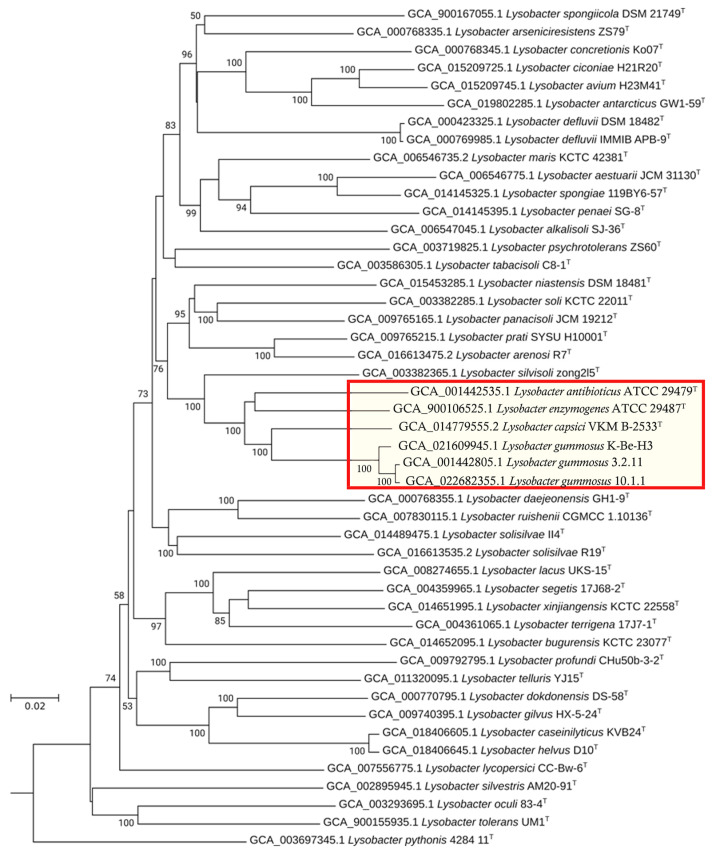
The phylogenomic tree of *Lysobacter*-type strains. The *Escherichia coli* O157:H7 genome sequence (assembly number, GCA_000008865.2) was used as an outgroup. The branching points indicate the values of their support greater than 50. The red box highlights the antimicrobial clade. The scale bar represents to 0.02 nucleotide substitutions per site.

In the first stage, we assessed the phylogenomic position of the strain *L. gummosus* 10.1.1 among the *Lysobacter* genus bacteria. For this purpose, the phylogenomic tree of the type strains was constructed (Figure 1). As can be seen in Figure 1, the species *L. gummosus* 3.2.11 and *L. gummosus* K-Be-H3 (the genome of type strain *L. gummosus* ATCC 29489 is not present in the open databases), as well as *L. capsici* VKM B-2533^T^, *L. enzymogenes* ATCC 29487T, and *L. antibioticus* ATCC 29479^T^, are genetically close to the strain *L. gummosus* 10.1.1.

Thus, *L. gummosus* is genetically close to the other species included in the genus *Lysobacter* at the time of its formation, as well as to the species *L. capsici*, which is known for its high antimicrobial potential. Altogether, these species form the antimicrobial clade of the genus *Lysobacter*.

**Figure 2:**
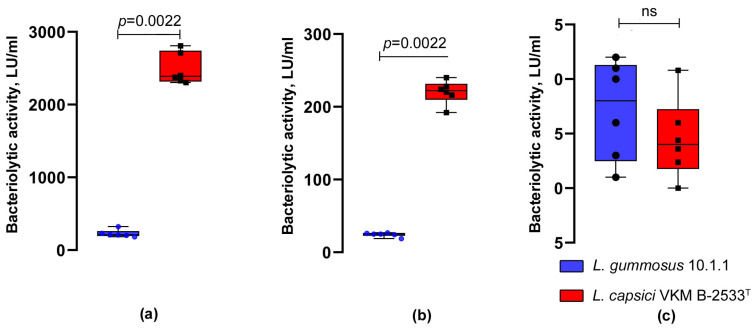
Comparison of the bacteriolytic activities of *L. gummosus* 10.1.1 and *L. capsici* VKM B-2533^T^. (**a**) Living cells of *M. luteus* Ac-2230^T^. The two groups were compared using the Mann–Whitney *U*-test. (**b**) Living cells of *S. aureus* 209P. The two groups were compared using the Mann–Whitney *U*-test. (**c**) Living cells of *B. cereus* 217. The two groups were compared using the unpaired two-tailed Student *t*-test. ns, the difference is statistically not significant.

To study the ability of *L. gummosus* 10.1.1 to lyse various target cells, the strain was cultivated on RM medium promoting the production of antimicrobial agents in *Lysobacter* bacteria. As a result of the cultivation, the culture fluid of the bacterium was found to have antimicrobial activity against living cells of *S. aureus* 209P, *M. luteus* Ac-2230^T^, and *B. cereus* 217 (Table 1, Figure 2). No lytic activity was revealed against Gram-negative bacteria *P. aeruginosa* and *P. vulgaris* H-19, as well as against mycelial fungi *F. solani* and *S. sclerotiorum*. For comparison, Table 1 shows data for the lytic activity of the culture fluid of the strain *L. capsici* VKM B-2533^T^, a well-known producer of antimicrobial agents [6]. It can be seen that the lytic activity of this strain against Gram-positive bacteria and fungi significantly exceeds that of strain *L. gummosus* 10.1.1. Only with respect to *B. cereus* do the activities of both strains coincide. Against Gram-negative bacteria, no lytic activity in the culture fluid of *L. capsici* was revealed, either.

Thus, *L. gummosis* 10.1.1 possesses an antimicrobial activity that can be due to its ability to produce antimicrobial agents of different natures, including bacteriolytic enzymes.

**Figure 3:**
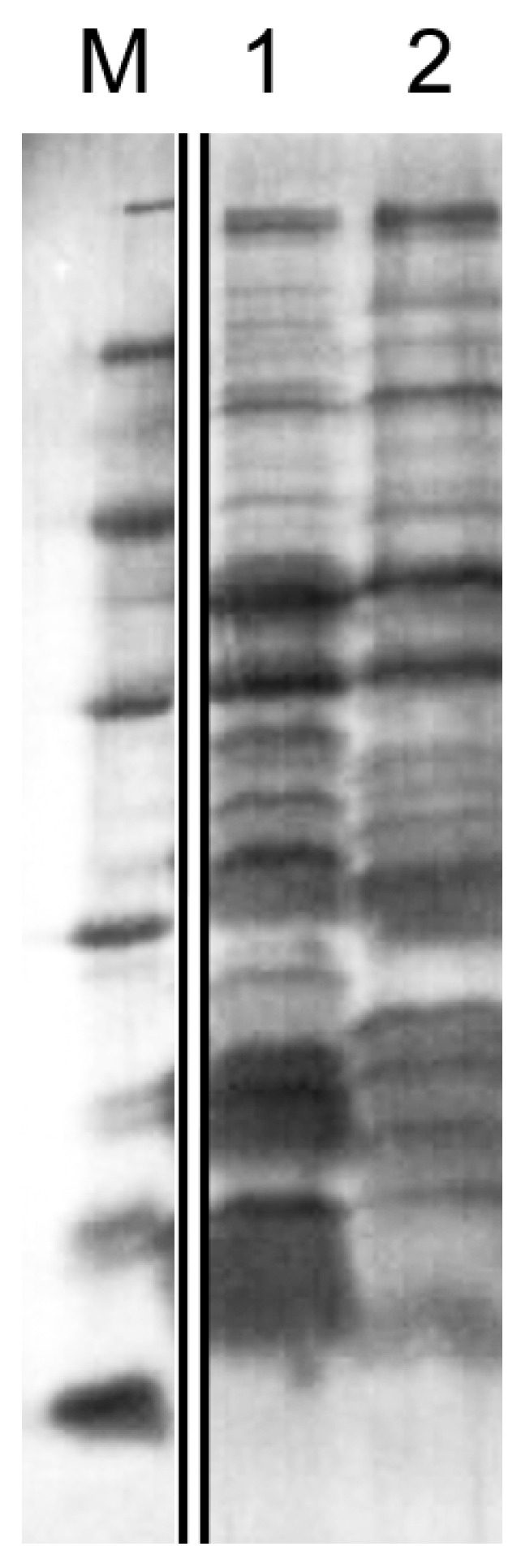
An electrophoregram of culture fluid proteins in SDS-PAGE. 1, culture fluid of *L. capsici* VKM B-2533^T^; 2, culture fluid of *L. gummosus* 10.1.1 (Supplementary File S1 Figure S1). Cultivation medium, RM.

The electrophoregram of the culture fluid proteins shows that *L. gummosus* 10.1.1 is an active producer of secreted proteins (Figure 3, lane 2). It can also be seen in the figure that the protein profile of strain 10.1.1 differs from that of strain VKM B-2533^T^ by the number of major proteins.

The total protein content in the culture fluid of *L. gummosus* 10.1.1 is 0.020 ± 0.001 mg/mL, which is 2 times less than in the culture fluid of *L. capsici* VKM B-2533^T^ (0.042 ± 0.003 mg/mL).

It is known that *Lysobacter* bacteria are capable of forming outer membrane vesicles that may contain antimicrobial agents [6,12,13]. For *L. gummosus* species, the ability to form vesicles has not been previously shown.

**Figure 4:**
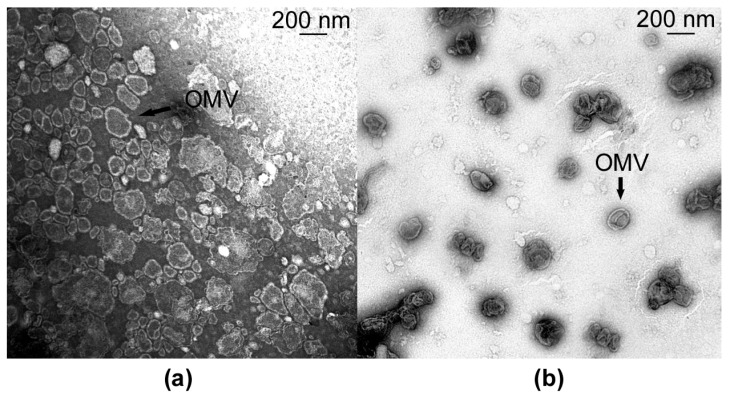
Electron microscopy of OMVs of *L. gummosus* 10.1.1 (**a**) and *L. capsici* VKM B-2533^T^ (**b**).

A preparation of vesicles was obtained from the culture fluid of strain 10.1.1 by differential centrifugation (Figure 4a). A preparation of *L. capsici* VKM B-2533^T^ vesicles was obtained for comparison (Figure 4b).

As can be seen in Figure 4a, the vesicle preparation of strain 10.1.1 contains intact vesicles 75 to 250 nm in diameter. Vesicles of strain 10.1.1 differ from those of strain VKM B-2533^T^ in diameter and morphology (Figure 4a,b). Vesicles of strain VKM B-2533^T^ are predominantly 170 nm in diameter (Figure 4b), whereas those of 100 nm predominate in the preparation of strain 10.1.1 (Figure 4a). Vesicles of strain 10.1.1 are polymorphic, often irregular in shape, in the form of elongated shapeless ovoids with sinuous edges. Those of strain VKM B-2533^T^ are of a more regular spherical shape.

It was noted that the vesicle precipitate of strain 10.1.1 obtained after ultracentrifugation was significantly less than that of strain VKM B-2533^T^. Absorption of the vesicle suspension in the preparation of strain 10.1.1 was OD_600_ = 1.0, whereas, in the preparation of vesicles of strain VKM B-2533^T^, it was OD_600_ = 4.0. This can indicate that *L. gummosus* formed fewer vesicles than *L. capsici* VKM B-2533^T^.

**Figure 5:**
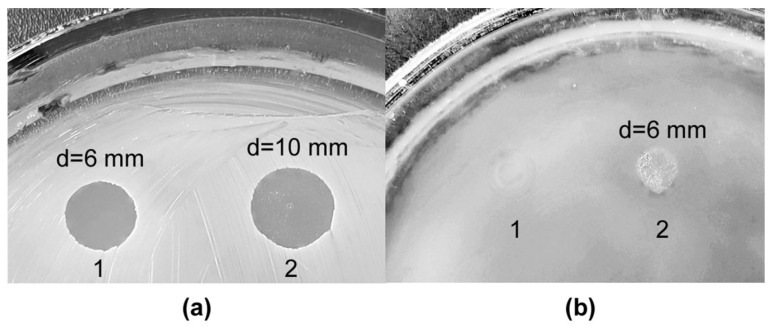
Bacteriolytic action of vesicles against living cells of *S. aureus* 209P (**a**) and *P. aeruginosa* (**b**). 1, Preparation of vesicles of *L. capsici* VKM B-2533^T^. 2, Preparation of vesicles of *L. gummosus* 10.1.1. d, Lysis zone diameter.

When studying the antimicrobial action, we found that the vesicle preparation of *L. gummosus* 10.1.1 had a strong lytic effect against living Gram-positive bacteria, as well as against the Gram-negative bacterium *P. aeruginosa* (Table 2, Figure 5).

It should be noted that the lytic action of vesicles of strain 10.1.1 against Gram-positive bacteria is comparable to that of *L. capsici* VKM B-2533^T^ vesicles (Table 2, Figure 5). Vesicles of strain 10.1.1 were even more effective against living *S. aureus* 209P cells than those of strain VKM B-2533^T^. It is important that vesicles of strain 10.1.1 lysed *P. aeruginosa* cells, whereas those of strain VKM B-2533^T^ had no such effect at all. Let us recall that no activity against *P. aeruginosa* was revealed in the culture fluid of both strains. This indicates that a lytic agent against *P. aeruginosa* is in the culture medium as part of *L. gummosus* 10.1.1 vesicles. The nature of this agent is to be determined in the future. No antifungal action of *L. gummosus* 10.1.1 vesicles was detected.

Thus, *L. gummosus* 10.1.1 has a pronounced antimicrobial potential, though not as strong as *L. capsici*. Nevertheless, the search for antimicrobial agents of *L. gummosus* 10.1.1 deserves attention; besides, there is an agent among them that lyses the living cells of *P. aeruginosa*.

### 3.2. Assessment of L. gummosus 10.1.1 Antimicrobial Potential via Transcriptomic Analysis

For transcriptomic analysis, *L. gummosus* 10.1.1 cells were cultivated on RM and 5/5 media for 19 h, which corresponded to the end of the exponential growth phase (Supplementary File S1 Figure S2). Cells grown on medium 5/5 were used as a control because in cultivation on this medium, we observed no lytic activity in the culture fluid of *L. gummosus* 10.1.1. After cultivation on the chosen media, the RNA was isolated from the cells and sequenced (Supplementary File S1 Figure S3).

The Illumina HiSeq 4000 platform generates an average of 12.6 million reads per sample. The lowest value is 8.3 million reads for a sample of RM rep. 1; the highest, 16.1 million reads for a sample of RM rep. 2 following the trimming by quality and adapter removal. All samples had sufficient sequencing depths, mostly greater than 10 million reads per sample. The average alignment rate to the reference genome of *L. gummosus* 10.1.1 was 95.3%, and 68.1% of reads, on average, were uniquely assigned to the annotated genes. All sequencing and alignment statistics are shown in Supplementary File S2.

Pearson *r*^2^ correlation values for all replicates were between 0.87 and 0.99, and the mean value for biological replicates was 0.99. A clustering tree of the samples also indicated the consistency of the obtained data. Analysis of differentially expressed genes (DEGs) revealed 497 genes upregulated and 470 genes downregulated at least 2 times compared to the control (*p*_adj_ < 0.05).

#### 3.2.1. Search for the Genes of *L. gummosus* 10.1.1 Bacteriolytic Enzymes

First of all, we searched for the genes of the bacteriolytic enzymes known for *Lysobacter*—these are bacteriolytic proteases L1, Blp, L4, L5, Serp, Serp3, Serp6, Serp7, and N-acetylglucosaminidase (Table 3).

Almost all genes we searched for were identified, with the exception of the gene of enzyme L5. It was found that in the cultivation of *L. gummosus* on RM medium, the expression of the genes of bacteriolytic enzymes Blp, L1, Serp3, Serp6, and Serp7 increased by 3.7, 8.6, 5.8, 5.1, and 2.7 times, respectively. Herewith, Blp and L1 are the key enzymes in the manifestation of bacteriolytic activity [6,32]. Only the expression of the Serp and L4 genes did not change, while that of the N-acetylglucosaminidase gene slightly decreased. On the whole, these results confirm once again that RM medium promotes the production of bacteriolytic enzymes.

Among bacteriolytic enzymes, the largest group is represented by proteases, which belong to the classes of serine proteases and metalloproteases. The genes of these proteases were searched for in *L. gummosus* 10.1.1; for them, an increase in the expression level was shown (Supplementary File S2). The genes that encode serine proteases proved to be 58%; the genes that encode metalloproteases, 36% (Supplementary File S1 Figure S4). First of all, we paid attention to the genes encoding metalloproteases of the M23 family (UNP30682.1), as well as serine proteases of the S1D family (UNP28310.1) (Table 3), because these families comprise the already known bacteriolytic enzymes. We also noted the genes encoding metalloproteases of the M4 family (UNP27383.1, UNP29729.1, UNP30981.1) and serine proteases of the S8 family (UNP29437.1, UNP29878.1). Almost all of these genes were shown to increase the expression level by more than twofold.

We also searched for the genes of bacteriolytic enzymes belonging to the group of amidases and glycosyl hydrolases. As a result, the gene of the enzyme UNP30261.1 was found, which is annotated as N-acetylmuramoyl-L-alanine amidase (Table 3). The expression level of this gene increased by 2 times. Among the glycosyl hydrolase genes, the GH25 family, to which lysozymes belong, is of the greatest interest. According to the CAZY database, only one gene of the UNP28866.1 enzyme was found in *L. gummosus* 10.1.1, which is annotated as the glycoside hydrolase family 25 protein. However, the expression level of this gene decreased (Supplementary File S2).

#### 3.2.2. Search for Genes of *L. gummosus* 10.1.1 Antifungal Enzymes

The genes of the antifungal enzymes β-1,3-glucanases GluA, GluB, GluC, and chitinase known for *Lysobacter* were also searched for [33,34]. The genes of these enzymes were identified in *L. gummosus* 10.1.1 (Table 3). An increase in the level of expression of these genes was shown. Moreover, the expression of the genes of the enzymes GluA and GluB increased significantly, by 199.5 and 347.7 times, respectively.

#### 3.2.3. Search for Genes Responsible for Biosynthesis of Antibiotics in *L. gummosus* 10.1.1

Production of antibiotics and antimicrobial peptides for bacteria of the genus *Lysobacter* has been shown earlier [5,11]. We searched for genes responsible for the biosynthesis of such compounds in *L. gummosus* 10.1.1 (Table 4).

As a result, we identified the genes responsible for the biosynthesis of antimicrobial agents known for *Lysobacter*. Noteworthy is the 116.3-fold increase in the expression level of the HSAF biosynthetic non-ribosomal peptide synthetase/polyketide synthase gene. HSAF is a well-investigated antifungal agent in *L. enzymogenes* [35,36,37,38]. A significant 22.2-fold increase in the expression level of the non-ribosomal peptide synthetase UNP29365.1 gene was also observed. An increase in the level of gene expression was also noted for non-ribosomal peptide synthetases UNP31811.1 and UNP31812.1. An increase in the expression level of the YcaO-like family protein gene was also noted.

Thus, transcriptomic analysis revealed the significant antimicrobial potential of *Lysobacter gummosus* 10.1.1; the gene products of the putative new antimicrobial agents deserve further study.

## 4. Discussion

*Lysobacter* bacteria are considered to be an inexhaustible source of various antimicrobial agents [39]. In the present work, we started studying the antimicrobial potential of *L. gummosus* 10.1.1. As a result of phylogenomic analysis, the genome of this strain was found to be clustered with the genomes of other lytically active species, which all together form an antimicrobial clade of the genus *Lysobacter*. The genome of *L. brunescens*, one of the first species that entered into the genus *Lysobacter* during its formation, is not included in this analysis [1]. Currently, there are no genomes available for this species in international databases. The species *L. silvisoli* [40], which is close to the active strains, is also of interest (Figure 1). However, there is currently no information about its lytic activity.

The present work established the antimicrobial effect of *L. gummosus* 10.1.1 culture fluid against living cells of *S. aureus* 209P, *M. luteus* Ac-2230^T^, and *B. cereus* 217. No activity against these bacteria has been shown earlier. Of interest were the results of research on the antimicrobial action of *L. gummosus* 10.1.1 vesicles. All Gram-negative bacteria form outer membrane vesicles [41,42]. However, not all vesicles possess an antimicrobial effect. Vesicles of strain 10.1.1 proved to lyse cells of both Gram-positive bacteria and of the Gram-negative pathogen *P. aeruginosa*. They were also active against *K. rosea* Ac-2200^T^, whereas the culture fluid was found not to have this activity. This can be explained by the diluted content of vesicles in the bacterium culture fluid. Against mycelial fungi, no activity was detected either in the culture fluid or in vesicles. For *Lysobacter* bacteria, the ability to form vesicles with antimicrobial action has been shown earlier. This ability significantly expands the spectrum of their antimicrobial action [4,6,12,13].

To date, the most lytically active species of the genus is *L. capsici*. To assess the antimicrobial potential of *L. gummosus*, we compared it with strain *L. capsici* VKM B-2533^T^. The antimicrobial potential of this strain has been sufficiently well investigated at our laboratory earlier [6]. A significant difference between *L. gummosus* 10.1.1 and *L. capsici* VKM B-2533^T^ is the complete absence of antifungal activity in it. Herewith, the antifungal activity of this strain has been shown previously [1,19]. In [19], the strain was cultivated for 2–3 days. By this time of cultivation, secondary metabolism genes become activated. Thus, the antifungal activity established for strain 10.1.1 can be due to the active production of secondary metabolites. In our experiments, cells of strain 10.1.1 were cultivated for 19 h, which corresponds to the end of the exponential growth stage, when secondary metabolism genes just begin to become activated, and secondary metabolites can be present in the culture fluid in small amounts. Nevertheless, by this time of cultivation, the antifungal activity in strain *L. capsici* VKM B-2533^T^ is quite pronounced. However, we cannot so far answer the question of what caused it—the production of bacteriolytic enzymes, antifungal enzymes, antibiotics, or their combined action.

Thus, *L. gummosus* 10.1.1 can be considered to be a promising producer of antimicrobial agents. A transcriptomic study was conducted to assess the lytic potential of its genome.

As a result of the transcriptomic analysis, almost all (eight) genes of the known bacteriolytic enzymes of *Lysobacter* were identified in the genome of strain 10.1.1, with the exception of the gene of bacteriolytic protease L5. These data were also compared with the earlier results of the transcriptomic analysis of *L. capsici* VKM B-2533^T^ [7] (Supplementary File S1 Table S2).

It is seen in Supplementary File S1, Table S2 that the expression levels of the genes of *L. capsici* bacteriolytic enzymes are considerably higher. This correlates with the bacteriolytic activity of the culture fluid of strain VKM B-2533^T^, which is also higher, and the spectrum of its antimicrobial action is wider. Thus, it can be assumed that the lower antimicrobial activity of strain 10.1.1 can be associated with the peculiarities of the regulation of the expression of bacteriolytic enzymes’ genes. This issue requires in-depth study.

A search was also performed for the genes of enzymes with putative bacteriolytic activity. First of all, we analyzed the genes whose expression increased and which are annotated as serine proteases and metalloproteases in accordance with the MEROPS database [43]. It is to these groups of enzymes that the well-known bacteriolytic proteases belong. As a result, in *L. gummosus* 10.1.1, we identified genes (Table 3) coding for metalloproteases UNP29729.1 and UNP30981.1 of the M4 family, which have no orthologs among the earlier isolated proteases. The gene of UNP27383.1 metalloprotease of the M4 family was also identified, which is 49.4% identical with a 79% coverage with the LasB (Q02RJ6) protease of *P. aeruginosa* UCBPP-PA14 [44]. This metalloprotease was also identified in the culture fluid of the type strain *L. gummosus* DSMZ 6980 [18]. The genes of serine proteases of the S8 (UNP29437.1, UNP29878.1) and S1D (UNP28310.1) families were identified. For serine protease of the S8 family, there are no orthologs among the earlier isolated proteases. Protease UNP28310.1 is 85% identical to *L. enzymogenes* protease 1 (P15636), with 95% coverage. Proteases UNP29878.1 and UNP28310.1 were also identified in the culture fluid of type strain *L. gummosus* DSMZ 6980 [18]. Recently, we have isolated serine protease Serp (UOF16681.1) of *L. capsici* VKM B-2533^T^, which also belongs to the S1D family, and it has been shown for the first time to be capable of hydrolyzing, in addition to protein substrates, autoclaved bacterial cells [7].

We also searched for bacteriolytic enzymes annotated as amydases. These enzymes hydrolyze the amide bond in the peptidoglycan of bacteria. A search for enzymes annotated as muramidases was carried out. Muramidases belong to glycosyl hydrolases of the GH25 family, which cleave the glycoside bond in the carbohydrate moiety of bacterial peptidoglycan. As a result, we found the gene of an enzyme annotated as N-acetylmuramoyl-L-alanine amidase (UNP30261.1). The gene of this enzyme is 94% identical to amidase CwhA (P81717) of *L. enzymogenes,* with 32% coverage. CwhA has been shown to be capable of hydrolyzing autoclaved bacterial cells [15]. Among muramidases, we found only the gene of the enzyme UNP28866.1, but the level of its expression decreased.

We also performed a search for *L. gummosus* 10.1.1 genes responsible for the biosynthesis of secondary metabolites (Table 4). It should be understood that the time of cultivation, chosen for transcriptome analysis, was determined by the interest in the production of bacteriolytic enzymes, not of secondary metabolites. For this reason, the latter were analyzed to a greater extent in order to establish their presence in *L. gummosus* 10.1.1. As a result, we identified the genes whose expression levels increased and which are responsible for the biosynthesis of an antibiotic with HSAF antifungal action [45], lanthipeptides [46], and non-ribosomal peptide synthetases that take part in the biosynthesis of the antibiotic lysobactin [47], as well as the genes of the YcaO-like family protein, which are involved in the modification of microcins [48].

A significant 116.5-fold increase in the expression level of the gene for the biosynthesis of the antifungal factor HSAF was noted. Herewith, the culture fluid and vesicles of *L. gummosus* 10.1.1 had no antifungal activity against the phytopathogenic fungi *F. solani* and *S. sclerotiorum*. It can be assumed that the HSAF biosynthetic pathway is not fully functioning by the time of strain 10.1.1’s cultivation. At the same time, in *L. capsici* VKM B-2533^T^ cultivated under the same conditions, the expression of the same HSAF biosynthesis gene increased by only 3.8 times. Herewith, the culture fluid and vesicles had strong antifungal action against these phytopathogens. Additional studies are required to understand these differences, including the analysis of the bacterial secretome, also at later stages of cultivation, as well as the isolation of appropriate agents in native form for their characterization. It should also be noted that both strains showed a significant increase in the expression levels of the genes of β-1,3-glucanases GluA, GluB, GluC, and chitinase, for which the antifungal activities are known (Table 3; Supplementary File S1, Table S2). However, at the moment, there is no sufficient information to discuss the role of these enzymes in the antifungal activities of *L. gummosus* 10.1.1 and *L. capsici* VKM B-2533^T^.

The antibiotic lysobactin has been first isolated from cells and culture fluid of *Lysobacter* sp. ATCC 53042 [47]. Lysobactin is predominantly active against Gram-positive bacteria and has insignificant activity against Gram-negative bacteria. *L. capsici* has no orthologs UNP31811.1 and UNP31812.1 involved in the biosynthesis of this antibiotic (Supplementary File S1 Table S3).

Both strains were noted to have an increase in the levels of expression of the YcaO-like family protein genes (Table 4; Supplementary File S1, Table S3). *L. capsici* VKM B-2533^T^ had as many as three orthologs of the UNP28784.1 protein belonging to this family. YcaO-like family proteins are involved in post-translational modification of the peptide chain, including in the biosynthesis of antibiotics, e.g., microcin B17 [48].

It was also of interest to observe an increase in the expression level of the non-ribosomal peptide synthetase UNP29365.1 of the *L. gummosus* 10.1.1 gene by 22.2 times (Table 4). *L. capsici* VKM B-2533^T^ has as many as three orthologs to this protein, and an increase in the expression level is observed for each gene of this protein (Supplementary File S1, Table S3). To date, the role of this synthetase is not known.

Both strains showed an increase in the levels of expression of the genes involved in the biosynthesis of lanthipeptides. It should be said that lanthipeptides have not been isolated from representatives of the genus *Lysobacter*. For lanthipeptides, antibacterial and antifungal activities are known [46].

Thus, *L. gummosus* 10.1.1 has significant antimicrobial potential, not inferior to that of *L. capsici*. However, this study revealed the urgent need to investigate the regulation of the expression of antimicrobial agent genes in *Lysobacter* bacteria. Solving this issue shall be the goal of our further research, along with the isolation and characterization of new enzymes with putative bacteriolytic activities.

## Figures and Tables

**Table 1:** Antimicrobial activities of the culture fluids of *L. gummosus* 10.1.1 and *L. capsici* VKM B-2533^T^.

Test Objects	Culture Fluid of *L. gummosus* 10.1.1, LU/mL	Culture Fluid of *L. capsici* VKM B-2533^T^, LU/mL
Bacteria *		
*M. luteus* Ac-2230^T^	227 ± 50	2488 ± 216
*S. aureus* 209P	24 ± 3	220 ± 16
*B. cereus* 217	27 ± 4	25 ± 4
*K. rosea* Ac-2200^T^	0	42 ± 5
*P. aeruginosa*	0	0
*P. vulgaris* H-19	0	0
Mycelial fungi **		
*F. solani*	–	++
*S. sclerotiorum*	–	++

* Bacteriolytic activities were determined by turbidimetry. ** Antifungal activities were determined by spot test.

**Table 2:** Antimicrobial activities of outer membrane vesicles of *L. gummosus* 10.1.1 and *L. capsici* VKM B-2533^T^.

Test Objects	OMVs of *L. gummosus* 10.1.1, d * (mm)	OMVs of *L. capsici* VKM B-2533^T^, d (mm)
Bacteria		
*M. luteus* Ac-2230^T^	10	9
*S. aureus* 209P	10	6
*B. cereus* 217	6	7
*K. rosea* Ac-2200^T^	11	10
*P. aeruginosa*	6	–
*P. vulgaris* H-19	–	–
Mycelial fungi		
*F. solani*	–	5
*S. sclerotiorum*	–	2.5

* Lysis zone diameter.

**Table 3:** Change of expression of the genes encoding the lytic enzymes of *L. gummosus* 10.1.1.

Enzymes	Change of Expression *
	(protein_id)/(locus_tag)
The known bacteriolytic enzymes of *Lysobacter*	
Protease Blp	3.7
	(UNP30460.1)/(MOV92_04075)
Protease L1	8.6
	(UNP31219.1)/(MOV92_08245)
Protease L4	ns
	(UNP27667.1)/(MOV92_14190)
Protease L5	No ortholog
Protease Serp	ns
	(UNP30471.1)/(MOV92_04130)
Protease Serp6	5.1
	(UNP29484.1)/(MOV92_23985)
Protease Serp7	2.7
	(UNP31934.1)/(MOV92_12050)
N-Acetylglucosaminidase	0.6
	(UNP30329.1)/(MOV92_03340)
Protease Serp3	5.8
	(UNP31461.1)/(MOV92_09555)
Enzymes with putative bacteriolytic activities	
N-Acetylmuramoyl-L-alanine amidase	2.0
	(UNP30261.1)/(MOV92_02985)
Serine protease S8	1.4
	(UNP29437.1)/(MOV92_23735)
Serine protease S8	3.8
	(UNP29878.1)/(MOV92_00900)
M4 family metallopeptidase	7.0
	(UNP27383.1)/(MOV92_12645)
M4 family metallopeptidase	2.9
	(UNP29729.1)/(MOV92_00120)
M23 family metallopeptidase	2.2
	(UNP30682.1)/(MOV92_05310)
M4 family metallopeptidase	2.0
	(UNP30981.1)/(MOV92_06955)
PKD domain-containing protein S1D	2.5
	(UNP28310.1)/(MOV92_17670)
Antifungal enzymes	
β-1,3-Glucanase GluA	199.5
	(UNP31829.1)/(MOV92_11495)
β-1,3-Glucanase GluB	347.7
	(UNP30940.1)/(MOV92_06720)
β-1,3-Glucanase GluC	5.3
	(UNP31788.1)/(MOV92_11290)
Chitinase	5.4
	(UNP31513.1)/(MOV92_09815)

* Values greater than unity correspond to an increase in expression; values smaller than unity correspond to a decrease in expression. ns, the level of expression did not change.

**Table 4:** Change of expression of the genes that can be involved in the biosynthesis of secondary metabolites in *L. gummosus* 10.1.1.

Proteins	Change of Expression *
	(protein_id)/(locus_tag)
Class III lanthipeptide	No ortholog
Class III lanthionine synthetase LanKC	No ortholog
Class 2 lanthipeptide synthetase LanM family protein	2.4
	(UNP31401.1)/(MOV92_09240)
Non-ribosomal peptide synthetase	22.2
	(UNP29365.1)/(MOV92_23340)
HSAF biosynthetic non-ribosomal peptide synthetase/polyketide synthase	116.3
	(UNP27785.1)/(MOV92_14840)
Non-ribosomal peptide synthetase (lysobactin)	2.0
	(UNP31811.1)/(MOV92_11405)
	3.4
	(UNP31812.1)/(MOV92_11410)
YcaO-like family protein	2.5
	(UNP28784.1)/(MOV92_20230)
Class 2 lanthipeptide synthetase LanM	1.9
	(UNP29075.1)/(MOV92_21820)
	1.3
	(UNP29632.1)/(MOV92_24800)

* Values greater than unity correspond to an increase in expression; values smaller than unity correspond to a decrease in expression.

## Data Availability

Data are contained within the article and Supplementary Materials.

## Supplementary Materials

The following supporting information can be downloaded at: https://www.mdpi.com/article/10.3390/microorganisms11122853/s1, Supplementary File S1, Figure S1: Original gel images for Figure 3; Supplementary File S1, Figure S2: Dynamics of growth and bacteriolytic activity during the cultivation of *L. gummosus* 10.1.1 on RM (a) and 5/5 (b) media; Supplementary File S1, Figure S3: Electrophoregram of the preparations of *L. gummosus* 10.1.1 total RNA in PAG with 8 M urea; Supplementary File S1, Table S1: RNA-Seq statistics; Supplementary File S1, Figure S4: Proteases whose genes increased their expression; Supplementary File S2: All sequencing and alignment statistics; Supplementary File S1, Table S2: Change of expression of the genes encoding the lytic enzymes of *L. capsici* VKM B-2533^T^; Supplementary File S1, Table S3: Change of expression of the genes that can be involved in the biosynthesis of secondary metabolites in *L. capsici* VKM B-2533^T^.
