# Supplementary material for: Lysobacter gummosus 10.1.1, a Producer of Antimicrobial Agents
Source: Microorganisms. 2023 Nov 24;11(12):2853. doi: 10.3390/microorganisms11122853 (PMC10745450; doi:10.3390/microorganisms11122853)
Supplement: Supplementary file 1 [file microorganisms-11-02853-s001.zip › Supplementary file S1.pdf]

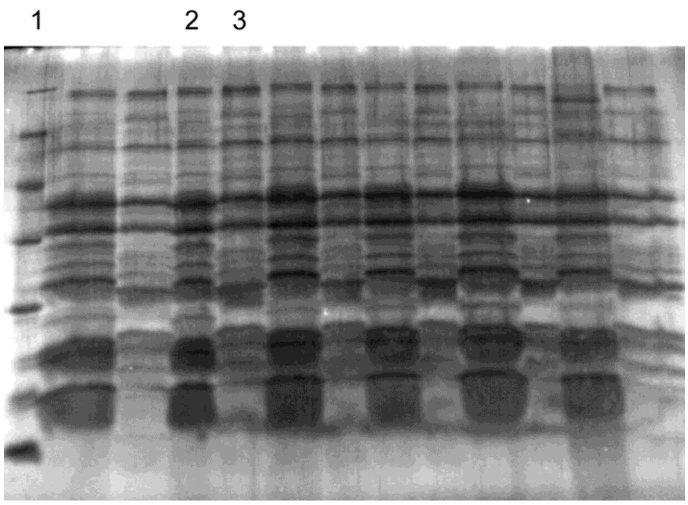

Supplementary File S1 Figure S1. Original gel images for Figure 3: lane 1 corresponds to M of Figure 3; lanes 2, to sample of culture fluid of *L. capsici* VKM B-2533^{T}; lanes 3, to sample of culture fluid of *L. gummosus* 10.1.1.

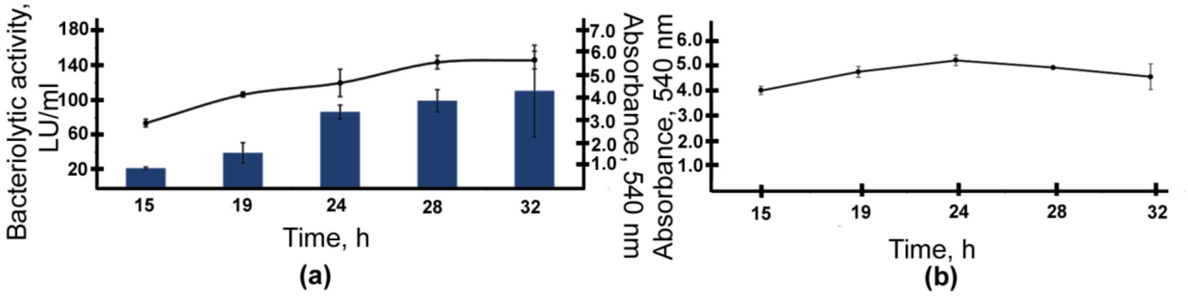

Supplementary File S1 Figure S2. Dynamics of growth and bacteriolytic activity during the cultivation of *L. gummosus* 10.1.1 on RM **(a)** and 5/5 **(b)** media.

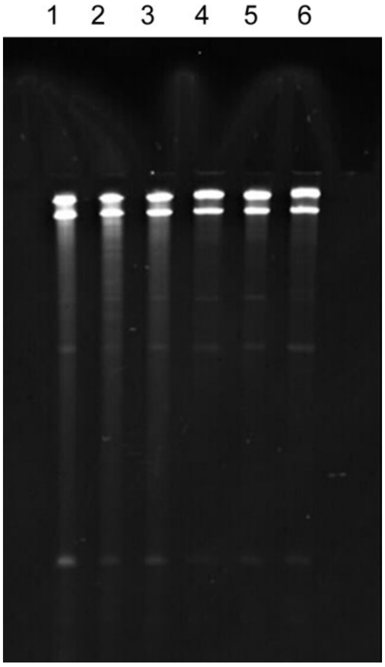

Supplementary File S1 Figure S3. Electrophoregram of the preparations of *L. gummosus* 10.1.1 total RNA in PAG with 8 M urea: 1, 2, 3 – 5/5 medium (3 biological replicates); 4, 5, 6 – RM medium (3 biological replicates). 100-ng RNA preparations each were loaded.

Supplementary File S1 Table S1. RNA-Seq statistics.

Sample	No. of reads before trimming	No. of reads after trimming	No. of mapped reads	No. of assigned reads
5-5 rep. 1	11,893,607	11,844,686	11,749,735	6,114,650
5-5 rep. 2	13,032,282	12,953,430	12,466,639	8,755,877
5-5 rep. 3	14,736,316	14,633,299	14,354,478	9,379,758
RM rep. 1	8,306,851	8,280,485	8,191,889	6,807,246
RM rep. 2	16,219,558	16,141,071	15,896,787	12,008,878
RM rep. 3	11,399,917	11,309,431	9,491,665	8,046,647

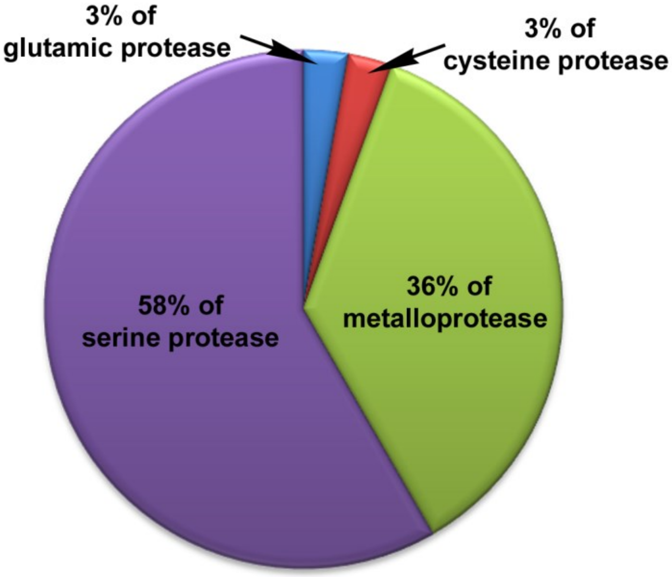

Supplementary File S1 Figure S4. Proteases whose genes increased their expression. The proteases were annotated using the Merops database (https://www.ebi.ac.uk/merops/).

Supplementary File S1 Table S2. Change of expression of the genes encoding the lytic enzymes of *L. capsici* VKM B-2533^T [7].

**Enzymes**	**Change of expression* (protein_id)/(locus_tag)**
The known bacteriolytic enzymes of *Lysobacter*	
Protease Blp	7.0 (UOF15870.1)/(IEQ11_04180)
Protease L1	39.1 (UOF13170.1)/(IEQ11_15580)
Protease L4	2.9 (UOF16892.1)/(IEQ11_09745)
Protease L5	6.0 (UOF13168.1)/(IEQ11_15570)
Protease Serp	3.2 (UOF16681.1)/(IEQ11_08595)
Protease Serp6	2.2 (UOF14691.1)/(IEQ11_23755)
Protease Serp7	4.2 (UOF17397.1)/(IEQ11_12530)
N-Acetylglucosaminidase	1.7 (UOF15745.1)/(IEQ11_03495)
Protease Serp3	8.1 (UOF12968.1)/(IEQ11_14490)
Antifungal enzymes	
β-1,3-Glucanase GluA	370.9 (UOF14439.1)/(IEQ11_22400)
β-1,3- Glucanase GluB	204.6 (UOF13513.1)/(IEQ11_17420)
β-1,3- Glucanase GluC	3.0 (UOF16369.1)/(IEQ11_06885)
Chitinase	14.5

	(UOF12917.1)/(IEQ11_14225)

*Values greater than unity correspond to an increase of expression; smaller than unity, to a decrease of expression.

Supplementary File S1 Table S3. Change of expression of the genes that can be involved in the biosynthesis of secondary metabolites in *L. capsici* VKM B-2533^{T} [7].

Proteins	Change of expression* (protein_id)/(locus_tag)
Class III lanthipeptide	7.7 (UOF16985.1)/(IEQ11_10290)
Class III lanthionine synthetase LanKC	8.5 (UOF16986.1)/(IEQ11_10295)
Class 2 lanthipeptide synthetase LanM family protein	1.7 (UOF15613.1)/(IEQ11_02800)
	5.2 (UOF15622.1)/(IEQ11_02845)
	3.1 (UOF14422.1)/(IEQ11_22315)
Non-ribosomal peptide synthetase	1.5 (UOF14553.1)/(IEQ11_23005)
	1.8 (UOF14554.1)/(IEQ11_23010)
HSAF biosynthetic non-ribosomal peptide synthetase/polyketide synthase	3.8 (UOF16764.1)/(IEQ11_09055)
non-ribosomal peptide synthetase (lysobactin)	No ortholog
YcaO-like family protein	3.2 (UOF14171.1)/(IEQ11_20965)
	5.9 (UOF14996.1)/(IEQ11_25355)
	2.7 (UOF14106.1)/(IEQ11_20620)
Class 2 lanthipeptide synthetase LanM	No ortholog

*Values greater than unity correspond to an increase of expression; smaller than unity, to a decrease of expression.
